# Complete remission after first-line radio-chemotherapy as predictor of survival in extranodal NK/T cell lymphoma

**DOI:** 10.1186/1756-8722-5-27

**Published:** 2012-06-08

**Authors:** Adrien Chauchet, Anne-Sophie Michallet, Françoise Berger, Isabelle Bedgedjian, Eric Deconinck, Catherine Sebban, Daciana Antal, Hubert Orfeuvre, Bernadette Corront, Tony Petrella, Maya Hacini, Marie Bouteloup, Gilles Salles, Bertrand Coiffier

**Affiliations:** 1 Department of Hematology, Centre Hospitalier universitaire Lyon Sud, Pierre Benite, France; 2 Department of Anatomopathology, Centre Hospitalier universitaire Lyon Sud, Pierre Benite, France; 3 Department of Anatomopathology, Besançon, France; 4 Department of Hematology, Centre Hospitalier universitaire Besançon, Besançon, France; 5 Department of Hematology, Centre Leon Berard, Lyon, France; 6 Department of Hematology, Centre Hospitalier de Roanne, Roanne, France; 7 Department of Hematology, Centre Hospitalier de Bourg en Bresse, Bresse, France; 8 Department of Hematology, Centre Hospitalier Anneçy, Anneçy, France; 9 Department of Pathology, Centre Hospitalier universitaire de Dijon, Dijon, France; 10 Department of Hematology, Centre Hospitalier de Chambery, Chambery, France; 11 Department of Hematology, Centre Hospitalier Lyon Sud, 165 chemin du grand revoyet, 69495, Pierre Bénite, France

**Keywords:** Extra nodal NK/T cell, Radiotherapy plus chemotherapy, Complete response, KPI index

## Abstract

****Background**:**

Extranodal nasal-type NK/T-cell lymphoma is a rare and severe disease. Considering the rarity of this lymphoma in Europe, we conducted a multicentric retrospective study on nasal-type NK/T cell lymphoma to determine the optimal induction strategy and identify prognostic factors.

****Methods**:**

Thirty-six adult patients with nasal-type NK/T-cell lymphoma were recruited and assessed. In total, 80 % of patients were classified as having upper aerodigestive tract NK/T-cell lymphoma (UNKTL) and 20 % extra-upper aerodigestive tract NK/T-cell lymphoma (EUNKTL).

****Results**:**

For advanced-stage disease, chemotherapy alone (CT) was the primary treatment (84 % *vs.* 10 % for combined CT + radiation therapy (RT), respectively), while for early-stage disease, 50 % of patients received the combination of CT + RT and 50 % CT alone. Five-year overall survival (OS) and progression-free survival (PFS) rates were 39 % and 33 %. Complete remission (CR) rates were significantly higher when using CT + RT (90 %) *versus* CT alone (33 %) (p < 0.0001). For early-stage disease, CR rates were 37 % for CT alone *versus* 100 % for CT + RT. Quality of response was significantly associated with survival, with 5-year OS being 80 % for CR patients *versus* 0 % for progressive disease patients (p *<* 0.01).

****Conclusion**:**

Early RT concomitantly or sequentially with CT led to improved patient outcomes, with quality of initial response being the most important prognosticator for 5-year OS.

## **Background**

Extranodal nasal-type NK/T-cell lymphoma is a rare and severe disease, occurring more frequently in Asia and South America than in Europe and North America [1-4]. This type of disease represents a distinct entity among T-cell lymphomas according to the World Health Organization (WHO) classification, [5] being found in both the nasal cavity and extranasal sites [6-8]. The disease is characterized histologically by the local invasion and necrosis of natural killer (NK) cells or T-cells with an invariable Epstein-Barr virus (EBV) infection. In published studies involving adults with a median age of 50 years, 60-90 % of lymphomas were localized in the nasal and upper airway regions, with the remaining found in extranasal sites [2,9,10]. Due to the low incidence of the disease, only a few randomized controlled trials have been undertaken [11-13]. Previous studies showed that 5-year overall survival (OS) rates were less than 40 %, with progression usually occurring within 2 years [2,7,9-15]. However, the early use of sequential radiotherapy (RT) and chemotherapy (CT) for localized nasal NK/T-cell [13,16] lymphoma was shown to be a successful therapy, which cured approximately half of patients [1,17]. In most patients with advanced disease (stage III/IV), the clinical course is highly aggressive, with frequent CT resistance and poor outcome. CHOP-based therapy [18,19] was often used, but associated without satisfactory results. However, recent combination therapies involving L-asparaginase have improved the outcome in high-risk, refractory, or relapsed patients [20-22]. The optimal therapy for advanced-stage or relapsed and refractory disease is yet to be established, although the results of several recent prospective trials showed improved results using CT or CT + RT [20,23-25]. Considering the rarity of this lymphoma in Europe, we conducted a multicentric retrospective study on nasal-type NK/T cell lymphoma. To this end, we reviewed the clinical and biological characteristics as well as treatments of 36 patients, with outcomes analyzed according to disease responses in order to determine the optimal induction strategy and identify prognostic factors.

## **Results**

### **Patient characteristics**

Our study included 36 patients with a median age of 49 years (range: 22 to 80), comprising 24 males and 11 females (ratio: 2.18:1). The majority of patients (75 %) were younger than 60 years. Among the 36 patients, 29 (80 %) were classified as upper aerodigestive tract NK/T cell lymphoma (UNKTL) *versus* 7 (20 %) patients as extra-upper aerodigestive tract NK/T cell lymphoma (EUNKTL). In addition, 72 % of UNKTL patients had local invasiveness greater than T3 with TNM classification. According to the Ann Arbor staging system, 10 (28 %) patients were categorized as stage I, six (17 %) as stage II, and 20 (55 %) as stage IV. All EUNKTL patients (n = 7) were considered to be stage IV. Furthermore, 75 % of patients had a good performance status (0–1). The most frequently involved sites were the nose (69 %), paranasal sinus (58 %), palate-pharyngeal (25 %), and bones (22 %). Regional lymphadenopathies were involved in 44 % of cases, bone marrow and central nervous system in 22 % and 11 %, respectively. The staging was based on computer tomography and MRI for most of the patients. Positron emission tomography/computed tomography (PET/SCAN) was not used to stage or follow evolution of the disease exept in recent diagnosis of T/NK lymphoma. The most frequent symptoms at diagnosis were obstructive in nature (purulent rhinorrhea, nasal obstruction, sinusitis, and dysphagia) in addition to epistaxis and cervical lymphadenopathy. Hematophagocytosis was observed in three patients. B symptoms were present in 39 % of patients. Overall, 14 (39 %) patients presented lactate dehydrogenase levels above the normal limit, 18 (50 %) high CRP levels, and 21 (58 %) increased beta2-microglobulin levels. Lymphopenia was found in 26 (61 %) patients, anemia in 13 (36 %), and low serum albumin level in 12 (33 %). According to IPI scoring, 23 (64 %) patients were classified as low-risk (0–2) and 13 (36 %) as high-risk. As a bone marrow biopsy was not performed in one patient, only 35 patients were assessed using PIT scores, revealing 24 (68 %) patients to be low-risk *versus* 11 (32 %) high-risk. According to Korean Prognostic Index (KPI) scoring, 19 (53 %) patients were considered low-risk compared to 17 (47 %) high-risk. Patient characteristics are listed in Table 1.

**Table 1:** Clinical characteristics of the 36 patients diagnosed with NK/T cell lymphoma 'nasal type'

**Number of patients**	36
**Median age (years ; range)**	49 (22–80)
**Age > 60 years, n (%)**	9 (25)
**Gender (male – female)**	24 – 11
**WHO performance status, n (%)**	
0- 1	27 (75)
2	6 (17)
3–4	3 (8)
**Local invasiveness in Upper NK / T lymphoma: T3 and T4, n (%)**	21 (72)
**Signs and symptoms, n (%)**	
Purulent rhinorrhea	20 (55)
Nasal obstruction or edema	25 (69)
Sinusitis	12 (33)
Epistaxis	11 (30)
Pharyngitis	5 (14)
Orbital edema or uveitis	5 (14)
Dysphagia	2 (5)
Nerve VII, palsy	2 (5)
**B symptoms, n (%)**	14 (39)
**Anatomic category, n (%)**	
Upper NK/T lymphoma	29 (80)
Extra upper NK/T lymphoma	7 (20)
**Sites of localization, n (%)**	
Nose	25 (69)
Paranasal sinus	21 (58)
Nodes	16 (44)
Palate – pharyngeal	9 (25)
Bone marrow	8 (22)
Orbit	4 (11)
Skin	4 (11)
Lung	4 (11)
Central nervous involvement	4 (11)
Liver	4 (11)
Gastrointestinal tract	3 (8)
Spleen	2 (5)
Testis	1 (3)
Suprarenal gland	1(3)
**Regional lymphadenopathy, n (%)**	16 (44)
**Biology, n (%)**	
LDH > Upper limit of normal	14 (39)
Lymphopenia	22 (61)
B2 microglobuline > upper limit of normal	21 (58)
CRP > upper limit of normal	18 (50)
Anemia	13 (36)
Serum albumin <30 mg/L	12 (33)
**Hemophagocytic syndrome, n (%)**	3 (8)
**Ann Arbor staging, n (%)**	
IE	10 (28)
IIE	6 (17)
IV	20 (55)
**IPI score, n (%)**	
0 – 1 (low risk)	16 (44)
2 (low intermediate risk)	7 (20)
3 (high intermediate risk)	7 (20)
4 -5 (high risk)	6 (16)
**Korean NK/T cell prognostic index, n (%)**	
0 (low risk)	5 (14)
1 (low intermediate risk)	14 (39)
2 (high intermediate risk)	8 (22)
3 – 4 (high risk)	9 (25)
**PIT score, n (%)**	
0 (low risk)	13 (37)
1 (low intermediate)	11 (31)
2 (high intermediate risk)	8 (22)
3 – 4 (high risk)	3 (8)

### **Treatment modalities**

The different first-line CT regimens are presented in Table 2. CT alone was the primary treatment for advanced-stage disease (84 % for CT *vs.* 10 % for CT + RT), with 82 % of patients receiving anthracycline-based regimens. For the 35 patients receiving CT, forty different protocols were used: anthracycline based regimen, high dose aracytine plus high dose methotrexate, high dose methotrexate plus L-asparaginase, cisplatin based regimen. For early-stage disease, an equal number of patients received CT + RT or CT alone (50 %-50 %). All seven patients with EUNKTL were administered CT alone. For patients with UNKTL, CT alone was given to 17 (61 %) patients compared to 10 (36 %) receiving the combination of CT + RT. Patients were treated with a median of 2 CT lines (range: 1 to 4). One patient was lost of view after 6 cycles of chemotherapy without evaluation of the disease response.

**Table 2:** First line treatment of the patients with T/NK cell lymphoma

	**Number of patients n (%)**	**Stage I -II n (%)**	**Stage IV n(%)**	**UNKTL n (%)**	**EUNKTL n (%)**
**First line therapy**	36 (100)	16 (45)	20 (55)	29 (80)	7 (20)
Chemotherapy alone	25 (69)	8 (50)	17 (85)	18 (62)	7 (100)
Chemotherapy + radiotherapy	10 (28)	8 (50)	2 (10)	10 (35)	0
Radiotherapy alone	1 (3)	0	1 (5)	1 (3)	0
Anthracycline-based regimen	26 (74)	11 (69)	15 (79)	20 (71)	6 (86)
High-dose methotrexate + high-dose aracytine regimen	4 (11)	2 (12)	2 (11)	3 (11)	1 (14)
High-dose methotrexate + L-asparaginase	2 (6)	1 (7)	1 (5)	2 (7)	0
Cisplatin-based regimen	3 (9)	2 (12)	1 (5)	3 (11)	0

UNKTL: upper aerodigestive NK/T cell lymphoma, EUNKTL : extra-upper aerodigestive NK/T cell lymphoma.

### **Radiation therapy and modalities**

Ten patients received CT + RT. The RT was given with a median dose of 40 gray (40 to 46). Only one patient received RT alone (30 gray) plus corticosteroids because of advaned age and comorbidities.

### **Response to treatment**

At the end of treatment, complete remission (CR) was observed in 48 % of patients, while the remaining 52 % experienced partial response or progressive disease. For the entire cohort, CR rates were 33 % for CT alone compared with 90 % for RT + CT.. In the CT group, there was no significant difference (p *=* 0.77) in CR rates according to the type of regimen: 52 % of patients achieved CR with an anthracycline-based regimen, 66 % with the combination of methotrexate and L-asparaginase, and 34 % with cisplatin-based regimens

For UNKTL patients, CR rates were only 50 % for CT alone versus 90 % for RT + CT.. However, UNKTL patients with CT + RT have more favorable prognostic parameters than CT alone patients: 20 % stage IV versus 61 %, 10 % of elevated LDH versus 44 %; but the same percentage of local invasiveness and regional lymphadenopathy involved (70 % vs 67 % and 40 % vs 44 %, respectively). One patient received RT alone, but experienced disease progression on this regimen.

Among the low stage group (stage I-II), the distribution between the two treatment modalities is balanced confirming the better outcome with the combination CT + RT. However in the stage IV patients, the difference in the distribution between CT and CT + RT could create a significant bias in favor of the CT + RT group.

For EUNKTL patients, all were treated with anthracycline-based regimens, resulting in a CR rate of 43 %. Responses to the different modalities are provided in Table 3.

**Table 3:** Response rate after first line therapy in patient with NK/T lymphoma

	**Response**	
	**CR (%)**	**PD (%)**
**All patients**		
**First line therapy**	48	52
Chemotherapy alone	33	67
Chemotherapy + radiotherapy	90	10
Radiotherapy alone	0	100
**Stage I and II**	69	31
Chemotherapy alone	37	63
Chemotherapy + radiotherapy	100	0
**Stage IV**	31	69
Chemotherapy alone	31	69
Chemotherapy + radiotherapy	50	50
Radiotherapy alone	0	100
**UNKTL**	50	50
Chemotherapy alone	29	71
Chemotherapy + radiotherapy	90	10
Radiotherapy alone	0	100
**EUNKTL**		
Chemotherapy alone	43	57

OR: overall response, PD: progressive disease, UNKTL: Upper NK/T Lymphoma, EUNKTL: Extra Upper NK/T Lymphoma 35 patients analyzed because 1 patient lost of view for response.

### **Overall survival and progression-free survival**

**Figure 1:**
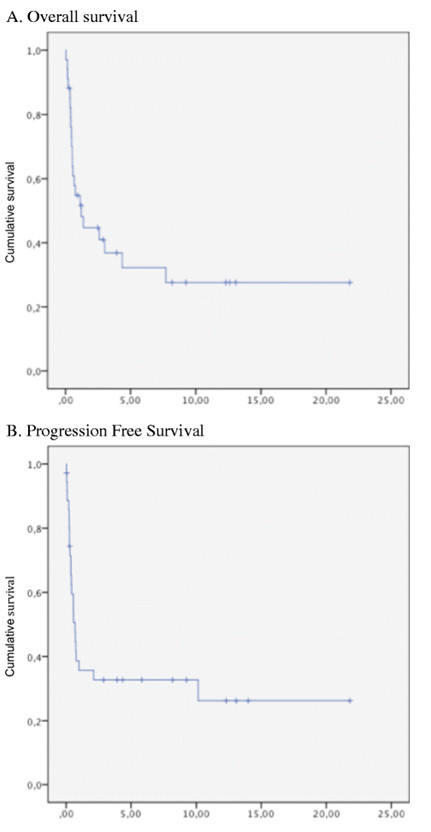
(**A**) **Overall survival (OS) for the entire cohort.** (**B**) Progression-free survival (PFS) for the entire cohort.

**Figure 2:**
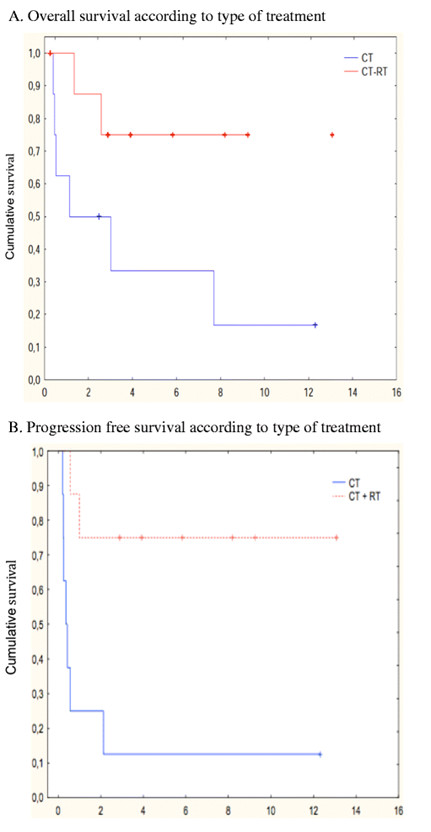
(**A**) **Overall survival according to type of treatment.** CT: chemotherapy; RT: radiotherapy; CT + RT: chemotherapy plus radiotherapy. (**B**) Progression-free survival according to type of treatment.

The 5-year OS and PFS rates were 39 % and 33 %, respectively (Figure 1A and Figure 1B). Eighty-two percent of patients died from disease progression, one from abdominal infection, and one from secondary acute leukemia 6 years after autologous transplantation. In univariate analysis, male sex, B symptoms, disease stage, lactate dehydrogenase level, prognostic indexes (IPI, PIT, and KPI), and quality of response (CR *versus* no response (or PD)) were found to be significantly associated with OS and PFS. Extensive disease stage, local invasiveness, high lactate dehydrogenase levels, and high-risk scores according to the IPI, PIT, and KPI classifications were related to poor survival. Quality of response was significantly associated with survival, with 5-year OS rates being 80 % for patients in CR versus 0 % for those with progressive disease (p *<* 0.01). When we focused on the UNKTL population, the best results were obtained when treatment consisted of CT + RT, with a statistically significant difference in terms of 5-year OS and PFS. The 5-year OS rate was 75 % for patients receiving CT + RT compared with 35 % for those receiving CT alone (p = 0.041) (Figure 2A). Accordingly, PFS was significantly higher in patients treated with RT + CT versus CT alone (p = 0.0063) (Figure 2B). Lymphopenia, low serum albumin level, high CRP level, and the classification into UNKTL or EUNKTL were statistically linked to OS or PFS.

## **Discussion**

Extranodal nasal-type NK/T-cell lymphoma is considered a distinct clinicopathological entity according to the WHO classification of lymphoid tumors, being more frequent in Asia and Central and South America than in Western countries [2,3,5,9,17,26]. In a recent international multicenter study reported by the International Peripheral T-cell Lymphoma Project, median survival time for patients with NK/T-cell lymphoma was 8 months, being the worst among all peripheral T-cell lymphomas included in the study [17]. To date, only a few studies have compared the clinical and pathologic features of patients with nasal or extranasal disease [7,10,17].

In our study, 36 French patients, all of European descent, observed over a 22-year period were diagnosed with nasal-type NK/T cell lymphoma..Main patient characteristics, such as age, sex ratio, B symptoms, and clinical aspects of the disease, were comparable to those previously reported [17,27,28]. At diagnosis, 80 % of tumors involved the upper aerodigestive tract, including the nasal and oral cavity, as well as the naso-, oro- and hypopharynx, while 20 % were extranasal. Disease presentation in our population was aggressive, with 65 % of UNKTL patients presenting stage IV at diagnosis. Although extranasal cases presented more adverse clinical features, no statistical differences were found in terms of OS or PFS between UNKTL and EUNKTL patients. This absence in OS difference may be accounted for by the more advanced disease stage of the UNKTL population. Previous studies reported bone marrow and central nervous system involvement at presentation to be rare, affecting 3 % and 7 % of cases, respectively [8,29-31]. In our study, however, bone marrow and central nervous system involvement was observed in 22 % and 11 % of patients, respectively. However, regional node involvement was found to be more frequent, affecting 44 % of patients [32,33]. In line with previous studies, we identified several variables associated with poor survival, notably regional lymph node involvement [34], local invasiveness [34-37], elevated lactate deshydrogenase [1,34], poor performance status [1], and B symptoms [1,37,38].

Several staging systems have been proposed in respect to nasal NK/T-cell lymphoma in order to predict prognosis. The Ann-Arbor staging system, while designed mainly for Hodgkin lymphoma, is not always accurate in the case of NK-cell lymphomas. In a number of studies on nasal NK-cell lymphoma, the involvement of areas outside the nasal cavity, including the paranasal sinuses, nasopharynx, and orbits, was defined as stage IE disease. In this case, the Ann-Arbor staging system cannot be used to determine the extent of the disease. Thus, for our patients, we scored local invasiveness greater than or equal to T3 according to the TNM classification. Overall, 72 % of UNKTL patients had a local invasiveness > T3, which was associated with worse survival.

Several studies investigated the impact of IPI in patients with nasal NK/T-cell lymphoma [7,9,13]. However, the use of this index has been controversial [13,34,39,40], as in a number of studies, only a small proportion of NK/T-cell lymphoma patients (up to 7 %) were categorized as high-risk [1,13,34,41,42]. Assigning a conventional IPI score appears to be of limited value, since most cases of NK/T-cell lymphomas are localized and result in a low score, despite survival being poor.

Lee *et al*. [9] developed the KPI, a prognostic model based on four risk factors: B symptoms, advanced stage, elevated lactate dehydrogenase levels, and involvement of regional lymph nodes. When analyzed according to the number of risk factors, the 5-year OS rate was 81 % for patients with no risk factors (score 0) and 7-15 % for those with three to four (scores 3–4). Our data validated the prognostic impact of the KPI, with 5-year OS rates being 60 % for patients with score 0 *versus* 17 % for those with score 4. This index appears more accurate than the IPI score in distinguishing high-risk groups, potentially being the most appropriate prognostic scoring system for patients with nasal-type NK/T lymphoma [43].

There are currently a few randomized trials evaluating the different therapeutic options for nasal-type NK/T-cell lymphomas. The majority of studies are retrospective in nature and almost all are conducted in the geographical areas where the tumor is prevalent. Most authors reported on the use of RT alone or combined with CT, with RT being associated with high remission rates and prolonged survival, mainly in the case of localized disease [34,40,44,45]. In a study of 82 patients with localized disease, early RT was shown to be the only independent prognostic factor, with 5-year OS being significantly better in patients receiving >54 Gy [46]. There appears to be a consensus that the optimal dose is 50 Gy, to be delivered to both the nasal cavity and sinuses. Nevertheless, concurrent CT may improve both local and systemic disease control. When comparing CT alone to RT alone or CT + RT, CR rates were significantly higher in patients treated with CT + RT [13,28,34,44,47]. In addition, two recent reports on CT + RT for localized disease (stage IE to IIE) showed improved results compared to the historical controls using RT alone [24,48].

In our study, the overall response rate was 48 %, with progressive disease observed in 52 % of patients and no partial response. Combined therapy comprising RT and anthracycline-based CT regimens was associated with higher CR rates and longer OS compared to CT or RT alone, even in advanced-stage disease (CR rate of 31 % *vs* 50 % for CT and CT + RT, respectively). However, the CT + RT cohort has a better prognostic profile than the CT population and this difference will create a significant bias in favor of CT + RT group, especially for this high stage group. No CR was recorded for RT alone, although this treatment was only administered to one elderly patient with a non optimal dose of 40 Gy. In the two published metaanalyses, first-line CT regimens involved CHOP, CHOP-like schedules, etoposide, ifosfamide, or cisplatin. In our study, however, the disease did not respond well to these combinations. The introduction of L-asparaginase-containing regimens led to further improvements, as most studies using asparaginase-based regimens in a relapsed or refractory setting reported response rates of around 50 %, with 5-year OS being 65 % (86 % for localized disease and 38 % for advanced stage) [20,22,25,48-50]. In our study, the different CT regimens were compared, with no statistical difference (but low numbers of patients in each type) found in CR rates according to the type of regimen. The use of L-asparaginase-based regimens in induction therapy did not improve CR rates, although only four patients were administered this regimen. Data regarding the use of L-asparaginase as first-line therapy is therefore still needed.

For all patients, 5-year OS and PFS rates were 39 % and 33 %, respectively. In addition, the quality of response after first-line treatment was found to be crucial for survival, with 5-year OS being 80 % in CR patients compared to 0 % in progressive disease patients.

## **Conclusions**

In our study, the early use of RT concomitantly or sequentially with CT was shown to improve patient outcome, especially in the case of localized disease, and even in patients presenting aggressive forms. Based on these positive results, patients with localized disease should be administered RT + CT. The use of L-asparaginase-based regimens as first-line therapy should be considered for patients with disseminated disease, although prospective trials are still needed to confirm improved survival rates associated with this therapeutic approach.

## **Patients and Methods**

### **Patients and staging evaluation**

Between January 1989 and September 2010, 36 patients were recruited in seven hematological centers in France. All the patients in this series been entirely of European descent (no patients were of Asia or south America descent). Patients were required to fulfill the WHO criteria for clinicopathologic diagnosis and classification of NK/T-cell lymphoma [5]. All biopsied tissues were critically reviewed. The following data was collected: gender, age, clinical characteristics (performance status and symptoms), biological parameters (lactate dehydrogenase, C-reactive protein [CRP], and serum albumin), radiological presentation, and histopathological reports describing angiocentricity, angioinvasion, necrosis zones, and polymorphism of individual cells. Immunohistochemical studies had to be positive for NK/T-cell markers, including CD2, CD3, or CD56 cytotoxic molecule (TIA-1 or granzyme B), and for EBV-encoded small RNA, and negative for B-cell markers, such as CD20 or CD79. Patients with blastic NK-cell lymphoma/leukemia, aggressive NK-cell lymphoma/leukemia, and unspecified peripheral T-cell lymphoma were excluded from the analysis. Scores for the International Prognostic Index [51] (IPI), Korean Prognostic Index (KPI) [9], and Prognostic Index for PTCL/NOS [52] (PIT) were calculated for all patients. We separately analyzed the survival of patients with upper aerodigestive tract NK/T-cell lymphoma (UNKTL) and extra-upper aerodigestive tract NK/T-cell lymphoma (EUNKTL) [1,27]. UNKTL included all lymphomas confined to the nasal cavity, nasopharynx, and upper aerodigestive tract, whereas lymphomas at all other sites were considered to be EUNKTL. Patients with primary lesions within the nasal cavity and secondary lesions in other organs were classified as UNKTL. Local invasiveness was defined in accordance with the 2002 TNM classification of the American Joint Committee on Cancer [9].

### **Outcomes and treatment strategies**

CT schedules, RT dosages, and chronological sequence of treatments were analyzed for each patient. In terms of CT, treatments were based on anthracycline, aracytine or cisplatin regimens (doxorubicin, cyclophosphamide, vindesine, bleomycin, and prednisone [ACVBP]) for 17 (49 %) patients; cyclophosphamide, doxorubicin, vincristine, and prednisone (CHOP) for 9 (25 %) patients; cyclophosphamide, vincristine, prednisone, doxorubicin, and methotrexate (COPADM) followed by cyclophosphamide, cytosine arabinoside, and etoposide (CYVE) for four (11 %) patients; etoposide, methylprednisolone, cytosine arabinoside, and cisplatin (ESHAP) and dexamethasone (DHAP) for 3 (8.3 %) patients; high-dose methotrexate plus L-asparaginase for 2 (6 %) patients. Autologous bone marrow transplantation was performed as first-line consolidation on one patient. The different treatment schedules are listed in Table 2. Studied outcomes were treatment response, progression-free survival (PFS), and OS, with treatment response being evaluated at the end of treatment according to the standardized response criteria of Cheson *et al.*[53].

Regarding RT treatment, the median dose was 40 grays (Gy). All patients received RT from a linear accelerator with 4 megavolt (MV), 6 MV or 10 MV photons to achieve dose homogeneity. Generally, the planning target volume included all macroscopic lesions, the paranasalsinuses, the nasopharynx, the upper gum, and the palate with adequate margins. Regardless of primary tumor localization, elective cervical lymph node irradiation was not delivered unless the neck was involved clinically. The most common field arrangement was two lateral opposing photons fields with supplementation between the medial canthus by appropriate energy of electron.

### **Statistical analysis**

OS and PFS were estimated using the Kaplan-Meier product-limit method. OS was measured from the date of diagnosis to death or last follow-up visit. Unadjusted Cox proportional hazards models were employed to make group comparisons for baseline and treatment characteristics and Kaplan –Meier curves were used to quantify the percentage of patients who were free of recurrence or those who stay alive over time. A p-value <0.05 was considered statistically significant, with two-sided significance tests used for all p-values.

## **Competing interest**

The author(s) indicated no potential conflicts of interest.

## **Authors’ contributions**

Conception and design: AC, A-SM and BC Provision of study materials or patients: all of the co authors Collection and assembly of data: AC and A-SM. Data analysis and interpretation: AC, A-SM and BC Manuscript writing: AC, A-SM, GS and BC. Final approval of the manuscript: All the co authors.
